# Multivariable models for advanced colorectal neoplasms in screen-eligible individuals at low-to-moderate risk of colorectal cancer: towards improving colonoscopy prioritization

**DOI:** 10.1186/s12876-021-01965-5

**Published:** 2021-10-18

**Authors:** Sanjay K. Murthy, Lilia Antonova, Catherine Dube, Eric I. Benchimol, Gregoire Le Gal, Richard Hae, Stephen Burke, Tim Ramsay, Alaa Rostom

**Affiliations:** 1 Present Address: Department of Medicine, University of Ottawa, Ottawa, Canada; 2 Division of Gastroenterology, The Ottawa Hospital, Ottawa, Canada; 3 Ottawa Hospital Research Institute, 501 Smyth Road, Unit W1212, Ottawa, ON K1H 8L6 Canada; 4 School of Epidemiology and Public Health, University of Ottawa, Ottawa, Canada; 5 Division of Gastroenterology, Hepatology and Nutrition, University of Toronto, Toronto, Canada; 6 The Hospital for Sick Children, Toronto, Canada; 7 Department of Family Medicine, University of Toronto, Toronto, Canada; 8 Department of Nephrology, McMaster University, Hamilton, Canada

**Keywords:** Colorectal cancer, Colonoscopy, Clinical prediction rule, Multivariable prediction model

## Abstract

**Background:**

Advanced colorectal neoplasms (ACNs), including colorectal cancers (CRC) and high-risk adenomas (HRA), are detected in less than 20% of persons aged 50 years or older who undergo colonoscopy. We sought to derive personalized predictive models of risk of harbouring ACNs to improve colonoscopy wait times for high-risk patients and allocation of colonoscopy resources.

**Methods:**

We characterized colonoscopy indications, neoplasia risk factors and colonoscopy findings through chart review for consecutive individuals aged 50 years or older who underwent outpatient colonoscopy at The Ottawa Hospital (Ottawa, Canada) between April 1, 2008 and March 31, 2012 for non-life threatening indications. We linked patients to population-level health administrative datasets to ascertain additional historical predictor variables and derive multivariable logistic regression models for risk of harboring ACNs at colonoscopy. We assessed model discriminatory capacity and calibration and the ability of the models to improve colonoscopy specificity while maintaining excellent sensitivity for ACN capture.

**Results:**

We modelled 17 candidate predictors in 11,724 individuals who met eligibility criteria. The final CRC model comprised 8 variables and had a c-statistic value of 0.957 and a goodness-of-fit p-value of 0.527. Application of the models to our cohort permitted 100% sensitivity for identifying persons with CRC and > 90% sensitivity for identifying persons with HRA, while improving colonoscopy specificity for ACNs by 23.8%.

**Conclusions:**

Our multivariable models show excellent discriminatory capacity for persons with ACNs and could significantly increase colonoscopy specificity without overly sacrificing sensitivity. If validated, these models could allow more efficient allocation of colonoscopy resources, potentially reducing wait times for those at higher risk while deferring unnecessary colonoscopies in low-risk individuals.

**Supplementary Information:**

The online version contains supplementary material available at 10.1186/s12876-021-01965-5.

## Introduction

Colorectal cancer (CRC) is the second most common cause of cancer-related death, accounting for 3–4% of all deaths in Canada and other developed nations [1, 2]. Moreover, the burden of CRC continues to rise due to an aging population that is living longer [1]. Therefore, reducing CRC incidence and related mortality have become major public health goals.

By virtue of its ability to accurately diagnose and treat colorectal neoplasms, colonoscopy is considered the gold standard test to evaluate individuals with signs or symptoms or other risk factors for CRC, and is often preferred to fecal occult blood testing (FOBT) for asymptomatic average-risk screening [3]. Colonoscopy has been shown to reduce the incidence of colorectal cancer (CRC) and CRC-related death [4, 5]. However, colonoscopy is also an invasive test that carries potential serious risks for patients and significant costs for society [6, 7]. Colonoscopy demands are also increasing in the context of an aging population and the expansion of population-based FOBT screening programs in persons over age 50, prolonging wait times for individuals at high risk of harboring CRC [8]. Therefore, it is imperative to develop effective ways to target colonoscopy resources to those that would obtain the greatest benefit.

The greatest protective effect of colonoscopy is derived from its ability to detect and remove high-risk adenomas (HRA) and to diagnose early stage CRC (at a curable stage), as these lesions have the greatest potential to progress to advanced incurable CRC [9, 10]. Conversely, low-risk adenomas often do not progress to CRC or do so over a long time (typically more than 10 years) [11]. Studies have shown that persons with HRA have a higher CRC-related mortality risk while those with LRA alone have a lower CRC-related mortality risk than members of the general population [12, 13]. Therefore, colonoscopy would ideally be targeted to individuals with CRC or HRA, collectively termed advanced colorectal neoplasms (ACNs). However, ACNs are presently detected in less than 20% of persons over age 50 undergoing either screening or diagnostic colonoscopy [14, 15], and less than 6% of those undergoing average-risk screening [16]. Therefore, most individuals are unnecessarily exposed to the risks and costs of colonoscopy, and these individuals further increase wait times for individuals who are potentially at higher risk.

Current risk stratification methods for colonoscopy consider a limited number of factors applied in isolation, guided by expert opinion [3, 17]. However, the predictive value of any single factor is low, leading to misallocation of resources. Algorithms that consider the collective contribution of multiple risk factors and protective factors are not readily available for use in clinical practice. Earlier efforts at developing such models by different groups have been hampered by either poor performance, model complexity, difficult to ascertain or overly complex variables or inappropriate patient selection, limiting their utility [18–26]. An important shortcoming of most models is the absence of prior colonoscopy or polypectomy as model variables [22–26], despite their significant impact on future CRC risk [4, 5, 12, 13, 27, 28].

Therefore, we sought to derive better performing prediction models that could be easily applied in clinical practice to discriminate which individuals over age 50 could stand to benefit from early colonoscopy based on the likelihood of harbouring ACNs.

## Methods

### Study cohort and data sources

This study was approved by the Ottawa Health Science Network Research Ethics Board. We studied consecutive individuals aged 50 years or older who underwent outpatient colonoscopy for perceived low-to-moderate risk indications at The Ottawa Hospital (Ottawa, Canada) between April 1, 2008 and March 31, 2012. The Ottawa Hospital is a tertiary care facility that provides inpatient and ambulatory endoscopy services to a catchment area of more than 1.2 million individuals in Eastern Ontario. Only the first complete colonoscopy for each person during this period was included.

We conducted a chart review in potentially eligible individuals aged 50 or older that were identified as having undergone colonoscopy in The Ottawa Hospital’s archived medical records to assess eligibility criteria and to collect information on potentially important predictors of ACNs to test in our multivariable models. We excluded individuals who underwent colonoscopy for one or more high-risk indications, including: (i) inflammatory bowel diseases (IBD); (ii) recognized hereditary CRC syndrome (i.e. Lynch syndrome, familial adenomatous polyposis, etc.); (iii) personal history of CRC; (iv) prior incomplete polypectomy; and (v) colorectal polyp or mass identified by diagnostic imaging or sigmoidoscopy. We also excluded persons who were referred for colonoscopy based on a positive FOBT, as this is an independent screening test that stratifies persons on need for colonoscopy. We further excluded individuals who underwent colonoscopy in the context of a hospital admission, as they may have had life-threatening indications for colonoscopy and often have suboptimal bowel preparations. We additionally excluded individuals with rare indications for colonoscopy, such as unexplained venous thromboembolism and cancer of unknown origin. Finally, we excluded individuals who did not have a high-quality colonoscopy (incomplete colonoscopy due to failure to reach the cecum or terminal ileum or to clear the colorectum of all observed polyps, or reported suboptimal bowel preparation), patients for whom the documentation did not allow accurate ascertainment of colonoscopy indications or neoplastic findings and persons with invalid health care registration numbers (which were necessary for linkage to provincial administrative datasets). We conducted a chart review in eligible persons to collect information on age, sex, comorbidities, colonoscopy indications and neoplastic findings during colonoscopy.

We linked these individuals deterministically to province-wide health administrative datasets for Ontario, Canada (held at IC/ES) to ascertain historical sociodemographic, clinical and health care utilization variables that could not be readily determined through chart review. IC/ES is a not-for-profit research institute encompassing a community of research, data and clinical experts, and a secure and accessible array of Ontario's health-related data [29]. The administrative datasets and variable coding definitions used in this study are provided in Additional file 1: Table S1. The accuracy of capture of colonoscopy procedures in the Ontario Health Insurance Plan (OHIP) database [30], of primary discharge diagnoses and procedures in the hospital discharge abstract database [31], and of cancer diagnoses in the Ontario Cancer Registry [32] have been shown to be high.

**Table 1:** Distribution of candidate predictors and outcomes in study cohort (N = 11,724)

Variable	Distribution
Major Colonoscopy Indication: Signs/symptoms	39.6%
Major Colonoscopy Indication: First-degree relative with CRC	24.9%
Major Colonoscopy Indication: Surveillance (history of polyps)	14.3%
Major Colonoscopy Indication: Asymptomatic screening*	28.2%
Symptom Indication: Rectal bleeding	19.7%
Symptom Indication: Anemia	7.4%
Symptom Indication: Unintentional weight loss	1.6%
Symptom Indication: Other gastrointestinal symptoms (abdominal or rectal pain, new diarrhea or constipation, rectal urgency, fecal incontinence)	20.3%
Age in years (median (IQR))	61 (55–68)
Male sex	46%
Charlson-Deyo co-morbidity score	
0–1	93.2%
2 +	6.8%
History of diabetes mellitus	17.6%
Prior gastrointestinal malignancy (excluding CRC)	0.5%
Any prior malignancy (excluding CRC)	11.1%
Lower endoscopy within past 10 years	42.3%
# Years since most recent lower endoscopy	
< 2	4.9%
2–4	9.6%
4–6	18.0%
6–8	6.7%
8–10	3.1%
> 10 (or none)	57.7%
Complete colonoscopy (to cecum) within past 10 years	37.9%
Polypectomy or polyp fulguration within past 10 years	15.6%
Highest neoplasia grade at colonoscopy	
Invasive cancer	1.5%
High-risk adenoma**	11.5%
Low-risk adenoma or normal	87%

*Default indication if the three other major colonoscopy indications (signs or symptoms, family history, surveillance) were negative

**≥ 3 adenomas, adenoma ≥ 1 cm or adenoma with villous/serrated/high-grade dysplastic features

Following linkage to IC/ES datasets, we excluded persons who did not have valid and continuous health care registration in Ontario for at least ten years preceding the index colonoscopy or whose primary residence was in a small geographic region in south-eastern Ontario where physicians do not routinely submit billing claims, as physician claims data were necessary to determine historical colonoscopy exposure. We also excluded individuals who had undergone colonoscopy or sigmoidoscopy at any facility in the province within one year preceding their index colonoscopy, as these individuals may have had undisclosed high-risk indications for repeat colonoscopy. We further linked patients to the validated IC/ES registries for Ontario citizens with IBD (Ontario Crohn’s and Colitis Cohort [33]) or cancer (the Ontario Cancer Registry) to identify and exclude any individuals with a history of IBD or CRC that were not identified through chart review.

From administrative datasets, we obtained information on co-morbidity burden (based on the Charlson-Deyo index [34]), lower endoscopy exposure within the preceding ten years, polyp treatment within the preceding ten years and cancer history (both gastrointestinal and non-gastrointestinal cancers) for the final study cohort using administrative data.

### Study variables

Three of the study investigators who are practicing adult gastroenterologists (SM, CD, AR) convened to develop an evidence-based list of variables that could potentially influence a person’s risk of developing ACNs. From this list, we retained variables that could be accurately determined through retrospective review of patients’ medical records and would also be easy to apply in an office setting as part of a clinical prediction model. We identified key variables encompassing colonoscopy indications, age, sex, comorbidity burden, cancer history, colonoscopy history and polypectomy history. Environmental factors, such as diet and smoking history, body mass index, physical activity and NSAIDs use, were not included due to incomplete reporting in patients’ records and perceived difficulty with quantifying an individual’s lifetime exposure for future application in a predictive model. Candidate predictors ascertained for testing in the multivariable models are provided in Table 1.

We classified neoplastic findings into the following three mutually exclusive categories: cancer (n = 173), HRA (n = 1,349) and insignificant (LRA or normal) (n = 10,202). HRA was defined as any of large adenoma (> 1 cm), multiple (≥ 3) adenomas or adenoma with any of villous, serrated or high-grade dysplastic features, as per established guidelines [17]. LRA was defined as 1–2 sub-centimeter tubular adenomas without high grade dysplasia.

### Model building and interpretation

We performed a complete case analysis for model building, given the large sample size and absence of any reason to suspect that cases with complete and incomplete information would differ systematically. We performed stepwise multivariable logistic regression modelling to arrive at our final models. All variables were tested for multicollinearity prior to inclusion. A candidate predictor could enter the model if its univariate association with the outcome was significant at a p-value of 0.2 and it was eliminated from the model if its independent association with the outcome was non-significant at a p-value of 0.1. Age was tested as a continuous variable; other variables were tested as categorical variables. We tested interaction terms between age, sex and prior colonoscopy exposure in the final models and retained any terms that were significant.

We first modelled CRC alone to ensure a high sensitivity of CRC capture (Model #1). We applied different sensitivity thresholds for CRC detection to determine probability cut-off points. Many individuals with HRA would inevitably be captured among persons deemed to require colonoscopy for CRC detection based on falling above a chosen probability threshold. Among persons who fell below the pre-specified thresholds in the CRC model, we derived a second model (Model #2) to capture residual ACNs (CRC or HRA not captured in the first model). We again applied various sensitivity thresholds for detection of ACNs to determine probability cut-off points, above which an individual would be deemed to require colonoscopy. We then evaluated different probability cut-off point pairs for the two models to determine the overall sensitivity and specificity for predicting CRC and HRA. We tested the robustness of our modelling strategy in the following subgroups of individuals: (i) those with signs/symptoms or other risk factors for CRC (all persons not undergoing asymptomatic average-risk screening); (ii) those with signs or symptoms (irrespective of other risk factors); (iii) those aged 50–74; and (iv) those aged 75 or older.

We assessed individual model performance by its discriminatory capacity (using the c-statistic value, equivalent to the area under the receiver operating curve) and calibration (using the Hosmer–Lemeshow goodness-of-fit test) [35]. We further assessed the ability of our model pair to increase the specificity of colonoscopy without overly sacrificing sensitivity for detecting ACNs at different probability cut points for the two models. We prioritized detection of CRC over detection of HRA in choosing optimal probability cut-off points, understanding that the latter could still be captured in a future screening exam before progressing to advanced CRC. We also tested different sensitivity thresholds for CRC and HRA detection, thereby allowing for flexible application of the models based on physician and patient preference as well as institutional/jurisdictional resources and priorities.

## Results

### Study cohort

**Fig. 1:**
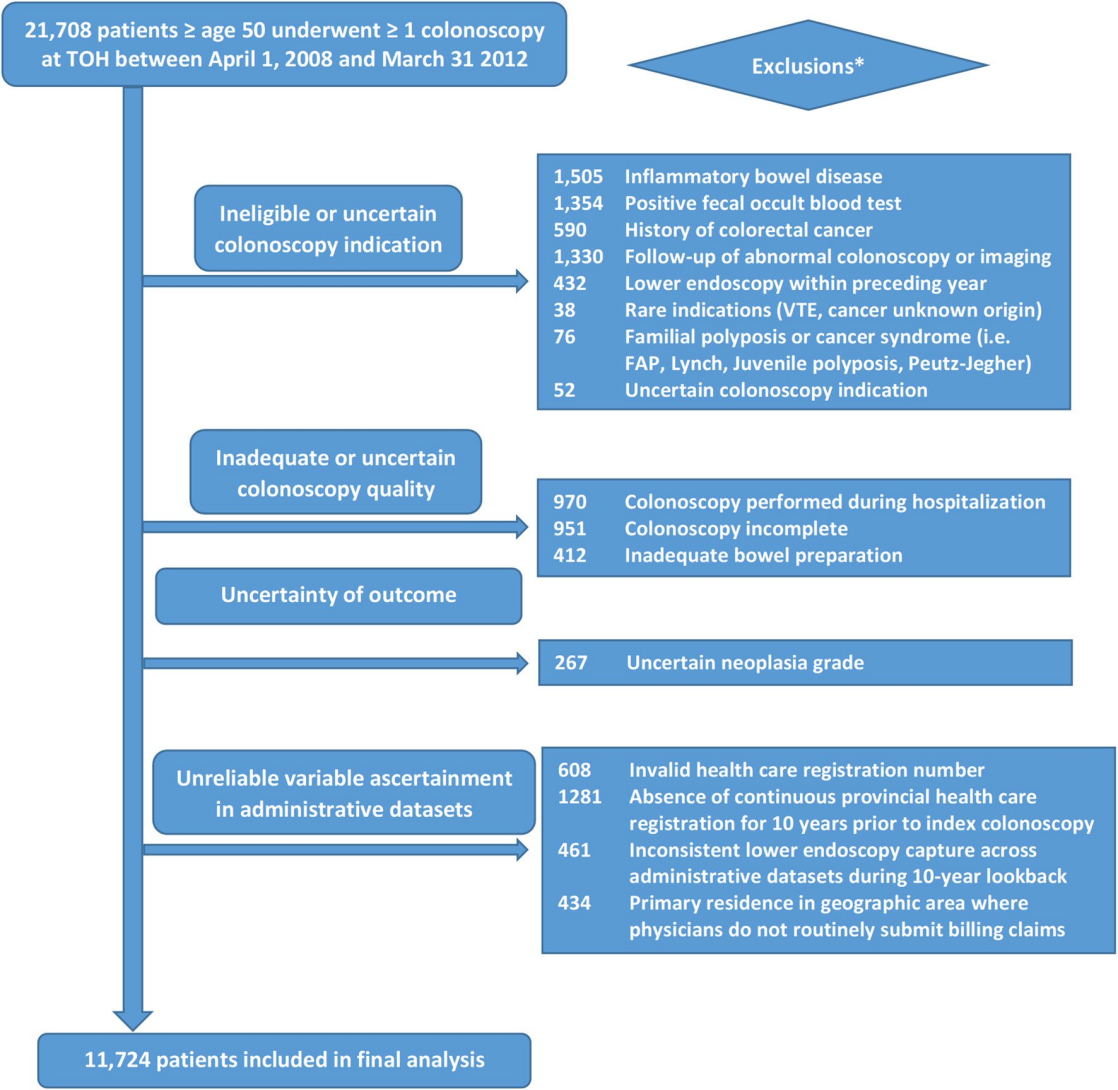
Study eligibility flow chart. *Patients could have had more than one exclusion criterion

Of 21,708 screened individuals aged 50 or older who underwent colonoscopy at TOH over the four-year study period, 11,724 met eligibility criteria. A flow chart of study exclusions is provided in Fig. 1. Of eligible individuals, 71.8% underwent colonoscopy for indications that would not constitute average risk screening and 13% had one or more ACNs identified during colonoscopy. The distribution of patient characteristics across candidate predictors and outcomes is provided in Table 2.

**Table 2:** Final model variables and model performance characteristics in complete study cohort

Variable	Estimate (SE)	Odds Ratio (95% CI)
*CRC model (Model #1)*		
Colonoscopy indication—signs/symptoms (y/n)	0.665 (0.286)	1.94 (1.11–3.40)
Colonoscopy indication—history of polyps (y/n)	0.687 (0.277)	1.99 (1.16–3.42)
Colonoscopy indication—rectal bleeding (y/n)	0.773 (0.232)	2.17 (1.38–3.41)
Colonoscopy indication—anemia (y/n)	0.578 (0.257)	1.78 (1.08–2.95)
Age (years)	0.0432 (0.00926)	1.04 (1.03–1.06)
Charlson-Deyo co-morbidity score (0–1 vs. 2 +)	2.33 (0.224)	10.3 (6.65–16.0)
History of non-CRC malignancy (y/n)	2.73 (0.245)	15.3 (9.45—24.7)
Complete colonoscopy within past 10 years (y/n)	− 1.66 (0.256)	0.191 (0.116–0.315)
Residual CRC or HRA model (Model #2)*		
Colonoscopy indication—CRC in FDR (y/n)	0.325 (0.114)	1.38 (1.11–1.73)
Colonoscopy Indication—history of polyps (y/n)	0.311 (0.158)	1.36 (1.001—1.86)
Age (years)	0.0384 (0.00783)	1.04 (1.02–1.06)
Sex (M/F)	− 0.470 (0.105)	0.625 (0.509–0.767)
# Years since any lower endoscopy		
1–2	− 0.0762 (0.219)	0.927 (0.604–1.42)
2–4	− 1.09 (0.216)	0.336 (0.22–0.513)
4–6	− 0.766 (0.168)	0.465 (0.334–0.646)
6–8	− 0.541 (0.207)	0.582 (0.388–0.873)
8–10	− 0.0903 (0.258)	0.914 (0.552–1.51)
> 10 or none	Ref	Ref
Polyp treatment within past 10 years (y/n)	0.794 (0.157)	2.21 (1.63–3.01)

c-statistic **0.957**

Goodness of fit p-value **0.527**

c-statistic **0.662**

Goodness of fit p-value **0.792**

*SE* standard error, *CI*  confidence interval

*Based on model of persons who fell below lowest probability cut point associated with 100% CRC sensitivity in Model #1

### Regression models and performance

**Fig. 2:**
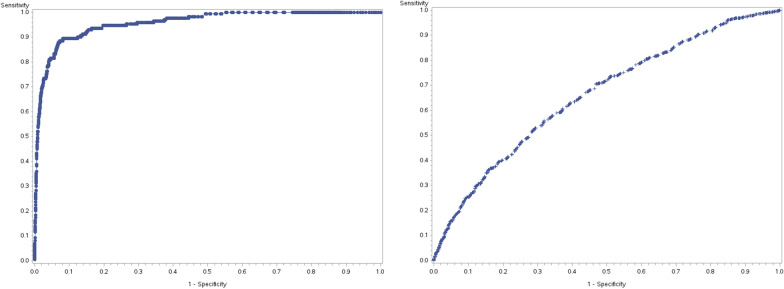
Receiver operating curves (ROC) for logistic regression models. Left panel—Model for CRC in complete cohort (AUROC 0.96). Right panel—Model for CRC/HRA among individuals falling below the minimum probability cut-off to permit > 99% CRC capture in Model #1 (AUROC 0.66)

The final variables retained in the models for CRC (Model #1) and residual ACNs (Model #2), along with model performance characteristics, parameter estimates and odds ratios are provided in Table 2. Receiver operating curves for each of these models are provided in Fig. 2. Cancer history, co-morbidity burden, colonoscopy history and age contributed substantially to overall model fit for Model #1, while colonoscopy indications contributed to a lesser degree (Table 2). Prior colonoscopy or polyp treatment, as well as age and sex, contributed substantially to overall model fit for Model #2, while colonoscopy indications contributed to a lesser degree (Table 2).

The calibration was good for both models in the overall cohort and in all subgroups (p-value > 0.5 for Hosmer–Lemeshow Goodness of Fit test in all cases). The regression model for CRC demonstrated excellent discriminatory capacity in the overall cohort and in all subgroups (c-statistic 0.95–0.96). The regression model for residual ACNs displayed modest discrimination (c-statistic 0.66–0.68 for all models).

The effects on colonoscopy specificity of applying our models to our reference cohort at various pre-determined sensitivity thresholds for CRC and HRA capture are provided in Tables 3 and 4 (complete cohort) and in Additional file 2: Table S2, Additional file 3: Table S3, Additional file 4: Table S4, Additional file 5: Table S5, Additional file 6: Table 6, Additional file 7: Table 7, Additional file 8: Table S8 and Additional file 9: Table S9 (subgroups). Applying both models in sequence to our reference cohort, the specificity of colonoscopy could be substantially improved with little loss in sensitivity for CRC and HRA detection (relative to performing colonoscopy in all individuals). For example, applying a probability cut point associated with 100% sensitivity for CRC detection in Model #1 permitted up to a 44% reduction in colonoscopy volume in our cohort (Table 3).

**Table 3:** Performance of logistic regression model of CRC at different sensitivity thresholds for CRC detection

Performance characteristic	Sensitivity of CRC detection		
	100%	99%	95%
% missed CRC	0	0.58	5.2
% missed HRA	31.5	35.7	68.2
% colonoscopies avoided	44.1	50.0	79.1

Interpretation (100% Column): At the minimum probability cut point to permit 100% CRC detection, the model predicts a miss rate of 0% for CRC and 31.5% for HRA and reduces colonoscopy burden by 44.1%

**Table 4:** Performance of sequential modelling strategy at different sensitivity thresholds for CRC and HRA detection

Performance characteristic	Sensitivity of CRC detection in complete cohort (Model #1)			Sensitivity of CRC or HRA detection in residual cohort (Model #2) (%)
	100%	99%	95%	
% missed CRC	0	0	0.58	80
% missed HRA	6.2	7.2	13.7	
% colonoscopies avoided	16.4	18.7	31.3	
% missed CRC	0	0.58	0.58	70
% missed HRA	9.5	10.5	20.7	
% colonoscopies avoided	23.8	25.5	40.9	
% missed CRC	0	0.58	0.58	60
% missed HRA	12.7	14.4	27.6	
% colonoscopies avoided	27.5	30.5	48.7	

Interpretation (100% Column, 80% row): At minimum probability cut points to permit 100% CRC detection in Model #1 and 80 CRC/HRA detection in Model #2, application of the sequential modelling strategy predicts a miss rate of 0% for CRC and 6.2% for HRA and reduces colonoscopy burden by 16.4%

Similarly, applying probability cut points in the sequential models (Models #1 and 2) to permit 100% sensitivity for CRC capture and greater than 90% sensitivity for HRA capture, allowed near 25% reduction in colonoscopy volume in our cohort (Table 4).

These findings were consistent across all subgroups, with application of the sequential models producing a minimum 20% reduction in colonoscopy volume with a miss rate of less than 1% for CRC and less than 10% for HRA (Additional file 2: Table S2, Additional file 3: Table S3, Additional file 4: Table S4, Additional file 5: Table S5, Additional file 6: Table S6, Additional file 7: Table S7, Additional file 8: Table S8 and Additional file 9: Table S9).

## Discussion

In this retrospective study of 11,724 consecutive individuals, 50 or older, who underwent colonoscopy for indications associated with a low-to-moderate risk of CRC, we derived multivariable prediction models comprising eight variables that would be easily ascertainable in clinical practice. Our models demonstrated excellent discriminatory capacity for CRC and good calibration for CRC and HRA, allowing for significant improvement in colonoscopy specificity in exchange for a small decrease in sensitivity of HRA capture. These findings were consistent across all designated subgroups of individuals. In particular, the contributions of less well-established risk factors, such as cancer history, comorbidity burden and patient sex, as well as of prior colonoscopy and polyp treatment, in our models provide a more complete and accurate picture of an individual’s current need for colonoscopy in the context of new signs or symptoms, family history of CRC or history of colorectal polyps.

Application of the models to our reference cohort would have permitted close to a 25% reduction in colonoscopy volume with no reduction in CRC detection and less than 10% reduction in HRA detection. It is conceivable that repeated application of the models at regular intervals (i.e. annually) could allow future capture of persons with HRA who were deemed to be at low risk by model criteria prior to their progression to advanced CRC. Notably, as large or advanced HRA will often give rise to signs or symptoms that would increase an individuals’ predicted probability of having CRC or HRA in our models, many of the HRA missed in applying our models are likely to be early HRA, such as 1–2 cm adenomas without high-grade dysplasia.

Importantly, the trade-off for a modest reduction in HRA capture through application of our models would be a substantial reduction in colonoscopy burden. This would then permit re-allocation of colonoscopy resources towards individuals predicted to be at higher risk based on model application or who have other higher risk indications, such as positive FOBT or IBD, while reducing unnecessary risks and costs of colonoscopy in many low-risk individuals. As judgements regarding an acceptable trade-off between missed advanced neoplasms and colonoscopy resource optimization will vary across patients, practitioners and settings, we have provided estimates for colonoscopy specificity at different sensitivity thresholds for CRC and HRA capture.

If successfully validated, our models have the potential to substantially improve colonoscopy prioritization over what is currently being offered. The ability to quantify an individual’s risk of harbouring CRC or HRA would enhance the process of patient-practitioner shared decision-making regarding the value and urgency of colonoscopy. We feel that our models would perform better than expert opinion and clinician judgment alone in persons with signs, symptoms or other risk factors for CRC (i.e. family history, previous polyps) and allow many persons who would otherwise be referred directly for colonoscopy to be redirected to undergo FOBT or else delay colonoscopy to a future time. Notably, the impact of our models on colonoscopy resource optimization ultimately depends on the frequency with which our models are applied in practice and with which the results are used to guide management.

To apply our models in clinical practice, a practitioner, typically a family physician or endoscopist, would ascertain values for the relevant model variables for a given patient and apply these to solve the model equation for probability of the individual harbour CRC in model#1 and residual ACNs in model #2, based on the desired level for sensitivity of lesion capture in the two models. As this would require advanced mathematical techniques, the process would be facilitated by an electronic application. It is to be noted that the purpose of our models differs from many prediction models, in that we are not interested in finding a probability cut point that optimizes sensitivity and specificity. We deemed sensitivity to be more important than specificity for diagnosing ACNs, particularly for CRC, and thereby selected higher yield sensitivity thresholds to improve colonoscopy specificity without overly sacrificing sensitivity of capture. For this reason, we also used a “two-model” strategy that allowed independent control of CRC ascertainment.

Furthermore, given the low prevalence of ACNs in our cohort (and society), the predictive capacity of our models for these outcomes (i.e. positive predictive value (PPV) and positive likelihood ratio (PLR)) is expected to be low at any cut point of predicted probability for CRC and HRA detection. Even at a specificity of 99% (sensitivity of 54%) for CRC in Model #1, the PPV is only 44%, and at 100% sensitivity, the PPV is just 2.6% (PLR 1.81). It is important for users of these models to remember that their purpose is not so bold as to definitively predict which individuals have ACNs, but to improve upon the specificity of ACN prediction over current methods. Therefore, in practice, our models should be used to “rule out” the possibility of an individual having CRC or HRA if they fall below a predetermined probability threshold associated with a high sensitivity of CRC and HRA capture, rather than to “rule in” an individual having ACNs if they fall above this probability threshold. The models would ultimately guide who could safely defer or avoid colonoscopy in this population.

Multiple groups have attempted to develop prediction models of ACNs, both in screening [18–21] and symptomatic [22–26] cohorts. However, shortcomings in model development have limited uptake of these models into clinical practice. Models that have been developed in asymptomatic average-risk screening cohorts have generally performed poorly. Models that have incorporated symptoms and other CRC risk factors have fared better, but have either not performed well enough for adoption to clinical practice practice [23, 24], have included too many variables to allow for easy application in clinical practice [23, 24, 26], have included variables that are challenging to ascertain and/or quantify in an office setting [23, 25], have included high-risk patient groups that would not be appropriate to risk stratify using such a tool [25, 26], or have focused on CRC prediction and ignored HRA [23–25]. In addition to overcoming most of these shortcomings, our models incorporated additional variables that have not been tested in most other models, including prior colonoscopy exposure and polyp treatment, as well as cancer history, all of which strongly influenced CRC and HRA risk prediction in our study.

Our study has several limitations. The reference cohort used to derive the models was ascertained retrospectively, which could have resulted in missing or inaccurate data for one or more variables, particularly colonoscopy indication. Due to our study population being restricted to the screen eligible people, our models cannot be extrapolated to persons under age 50. Moreover, our models did not test environmental risk factors for CRC, such as smoking, diet or NSAIDs because of the difficulty in ascertaining and quantifying the lifetime contribution of such factors. Finally, as these models have not been externally validated, they are not yet suitable for translation to clinical practice.

## Conclusions

In conclusion, we have been able to produce multivariable models with good calibration and excellent ability to discriminate individuals with and without ACNs, among those aged 50 or older who have perceived low-to-moderate CRC risk.. Our models are easy to apply in an office setting and could be used by general practitioners and endoscopists to enhance shared decision-making with patients regarding the utility of colonoscopy, afford a reduction in colonoscopy burden in lower risk individuals, and permit reallocation of colonoscopy resources towards individuals predicted to be at higher risk through model application or who have other higher risk indications. Our models will require external validation and translation to an electronic application prior to being suitable for clinical use.

## Supplementary Information

**Additional file 1.** **Table S1.** Ontario Health Administrative Databases and variable ascertainment codes for candidate predictors ascertained through administrative data.

**Additional file 2.** **Table S2.** Model performance at different sensitivity thresholds for CRC detection among patients with major CRC risk factors (CRC model only).

**Additional file 3.** **Table S3.** Model performance at different sensitivity thresholds for CRC and HRA detection among patients with major CRC risk factors (sequential models for CRC and residual ACNs).

**Additional file 4.** **Table S4.** Model performance at different sensitivity thresholds for CRC detection among patients with signs or symptoms (CRC model only).

**Additional file 5.** **Table S5.** Model performance at different sensitivity thresholds for CRC and HRA detection among patients with signs or symptoms (sequential models for CRC and residual ACNs).

**Additional file 6.** **Table S6.** Model performance at different sensitivity thresholds for CRC detection among patients aged 50–74 (CRC model only).

**Additional file 7.** **Table S7.** Model performance at different sensitivity thresholds for CRC and HRA detection among patients aged 50–74 (sequential models for CRC and residual ACNs).

**Additional file 8.** **Table S8.** Model performance at different sensitivity thresholds for CRC detection among patients aged 75 and older (CRC model only).

**Additional file 9.** **Table S9.** Model performance at different sensitivity thresholds for CRC and HRA detection among patients aged 75 and older (sequential models for CRC and residual ACNs).

## Data Availability

The datasets used and/or analyzed during the current study are available from the corresponding author on reasonable request.

## Abbreviations

CRC: Colorectal cancer
FOBT: Fecal occult blood test
HRA: High-risk adenomas
LRA: Low-risk adenomas
ACN: Advanced colorectal neoplasms
IBD: Inflammatory bowel disease
OHIP: Ontario Health Insurance Plan
PPV: Positive predictive value
PLR: Positive likelihood ratio
