# Supplementary material for: Multivariable models for advanced colorectal neoplasms in screen-eligible individuals at low-to-moderate risk of colorectal cancer: towards improving colonoscopy prioritization
Source: BMC Gastroenterol. 2021 Oct 18;21:383. doi: 10.1186/s12876-021-01965-5 (PMC8524805; doi:10.1186/s12876-021-01965-5)
Supplement: Supplementary file 1 — Additional file 1. Table S1. Ontario Health Administrative Databases and variable ascertainment codes for candidate predictors ascertained through administrative data. [file 12876_2021_1965_MOESM1_ESM.docx]

| **Supplemental** **Table 1:** Ontario Health Administrative Databases and Variable Ascertainment Codes for Candidate Predictors Ascertained Through Administrative Data^£^ | | |
| --- | --- | --- |
| **Variable** | **Database** | **Variable Ascertainment** |
| Charlson-Deyo comorbidity index | Canadian Institutes of Health Information Discharge Abstract Database and Same Day Surgery Databse (Ontario versions) | ICES macro using 2-year lookback for health care contacts with relevant diagnoses |
| Prior colonoscopy  Prior polypectomy or polyp fulguration | Ontario Health Insurance Plan (OHIP) database | Any lower endoscopy – any of the fee codes Z555A, Z580, Z496A, Z497A, Z498A or Z499A +/- any E codes  Complete colonoscopy - [any of Z555A, Z580, Z496A, Z497A, Z498A or Z499A] AND [E747A or E705A]  Polypectomy –any of Z571A, E720A, E685A, Z570A, E719A in conjunction with any lower endoscopy fee code |
| Diabetes Mellitus | Ontario Diabetes Database | Validated case ascertainment definition^1^ |
| Inflammatory Bowel Diseases | Ontario Crohn’s and Colitis Cohort | Validated case ascertainment definitions^2,3^ |
| Any Cancer  Colorectal Cancer | Ontario Cancer Registry | Diagnosis of any solid organ cancer  Diagnosis of colorectal cancer |
| £ ICES data dictionary available at: <https://datadictionary.ices.on.ca/Applications/DataDictionary/Default.aspx>  1 Lipscombe LL, Hwee J, Webster L, Shah BR, Booth GL, Tu K. Identifying diabetes cases from administrative data: a population-based validation study. *BMC Health Serv Res*. 2018;18(1):316. Published 2018 May 2. doi:10.1186/s12913-018-3148-0  2,3 Benchimol EI, Guttmann A, Griffiths AM, et al. Increasing incidence of paediatric inflammatory bowel disease in Ontario, Canada: evidence from health administrative data. Gut. 2009;58(11):1490-1497. Benchimol EI, Guttmann A, Mack DR, et al. Validation of international algorithms to identify adults with inflammatory bowel disease in health administrative data from Ontario, Canada. JClinEpidemiol. 2014;67(8):887-896. | | |
