# Supplementary material for: Multivariable models for advanced colorectal neoplasms in screen-eligible individuals at low-to-moderate risk of colorectal cancer: towards improving colonoscopy prioritization
Source: BMC Gastroenterol. 2021 Oct 18;21:383. doi: 10.1186/s12876-021-01965-5 (PMC8524805; doi:10.1186/s12876-021-01965-5)
Supplement: Supplementary file 2 — Additional file 2. Table S2. Model performance at different sensitivity thresholds for CRC detection among patients with major CRC risk factors (CRC model only). [file 12876_2021_1965_MOESM2_ESM.docx]

| **Supplemental Table 2. Model Performance at Different Sensitivity Thresholds for CRC Detection Among Patients with Major CRC Risk Factors***  **(CRC Model Only)** | | | |
| --- | --- | --- | --- |
| **Performance Characteristic** | **Sensitivity of CRC Detection** | | |
|  | **100%** | **99%** | **95%** |
| % missed CRC | 0 | 1.3 | 5.0 |
| % missed HRA | 33.7 | 42.7 | 70.8 |
| % colonoscopies potentially avoided | 46.4 | 56.0 | 80.0 |

^* signs/symptoms, first-degree relative with CRC or prior history of polyps^

Example of Interpretation (100% Column):

At 100% sensitivity threshold for CRC detection, application of the primary model for CRC would lead to a miss rate of 0% for CRC and 33.7% for HRA while permitting avoidance of up to 46.4% of colonoscopies
