# Supplementary material for: Multivariable models for advanced colorectal neoplasms in screen-eligible individuals at low-to-moderate risk of colorectal cancer: towards improving colonoscopy prioritization
Source: BMC Gastroenterol. 2021 Oct 18;21:383. doi: 10.1186/s12876-021-01965-5 (PMC8524805; doi:10.1186/s12876-021-01965-5)
Supplement: Supplementary file 3 — Additional file 3. Table S3. Model performance at different sensitivity thresholds for CRC and HRA detection among patients with major CRC risk factors (sequential models for CRC and residual ACNs). [file 12876_2021_1965_MOESM3_ESM.docx]

| **Supplemental Table 3. Model Performance at Different Sensitivity Thresholds for CRC and HRA Detection Among Patients with Major CRC Risk Factors***  **(Sequential Models for CRC and Residual ACNs)** | | | | |
| --- | --- | --- | --- | --- |
| **Performance Characteristic** | **Sensitivity of CRC Detection (Model #1)** | | | **Sensitivity of CRC or HRA Detection in Residual Cohort (Model #2)** |
|  | **100%** | **99%** | **95%** |  |
| % missed CRC | 0 | 0 | 0 | **80%** |
| % missed HRA | 6.7 | 8.5 | 14.4 |  |
| % colonoscopies potentially avoided | 19.9 | 23.5 | 33.5 |  |
| % missed CRC | 0 | 0 | 0 | **70%** |
| % missed HRA | 10.1 | 12.6 | 21.4 |  |
| % colonoscopies potentially avoided | 24.8 | 30.2 | 40.8 |  |
| % missed CRC | 0 | 0 | 0 | **60%** |
| % missed HRA | 13.4 | 17.2 | 28.6 |  |
| % colonoscopies potentially avoided | 28.9 | 35.1 | 49.9 |  |

^* signs/symptoms, first-degree relative with CRC or prior history of polyps^

Example of Interpretation (100% Column):

At 100% sensitivity threshold for CRC detection in Model #1 (entire cohort) and 80% sensitivity for CRC or HRA detection in Model #2 (residual cohort), the sequential modelling strategy would lead to a miss rate of 0% for CRC and 6.7% for HRA while permitting avoidance of up to 19.9% of colonoscopies
