# Supplementary material for: Multivariable models for advanced colorectal neoplasms in screen-eligible individuals at low-to-moderate risk of colorectal cancer: towards improving colonoscopy prioritization
Source: BMC Gastroenterol. 2021 Oct 18;21:383. doi: 10.1186/s12876-021-01965-5 (PMC8524805; doi:10.1186/s12876-021-01965-5)
Supplement: Supplementary file 4 — Additional file 4. Table S4. Model performance at different sensitivity thresholds for CRC detection among patients with signs or symptoms (CRC model only). [file 12876_2021_1965_MOESM4_ESM.docx]

| **Supplemental Table 4. Model Performance at Different Sensitivity Thresholds for CRC Detection Among Patients with Signs or Symptoms**  **(CRC Model Only)** | | | |
| --- | --- | --- | --- |
| **Performance Characteristic** | **Sensitivity of CRC Detection** | | |
|  | **100%** | **99%** | **95%** |
| % missed CRC | 0 | 0.7 | 5 |
| % missed HRA | 17.6 | 52.6 | 67.1 |
| % colonoscopies potentially avoided | 31.6 | 64.0 | 75.4 |

Example of Interpretation (100% Column):

At 100% sensitivity threshold for CRC detection, application of the primary model for CRC would lead to a miss rate of 0% for CRC and 17.6% for HRA while permitting avoidance of up to 31.6% of colonoscopies
