# Supplementary material for: Multivariable models for advanced colorectal neoplasms in screen-eligible individuals at low-to-moderate risk of colorectal cancer: towards improving colonoscopy prioritization
Source: BMC Gastroenterol. 2021 Oct 18;21:383. doi: 10.1186/s12876-021-01965-5 (PMC8524805; doi:10.1186/s12876-021-01965-5)
Supplement: Supplementary file 5 — Additional file 5. Table S5. Model performance at different sensitivity thresholds for CRC and HRA detection among patients with signs or symptoms (sequential models for CRC and residual ACNs). [file 12876_2021_1965_MOESM5_ESM.docx]

| **Supplemental Table 5. Model Performance at Different Sensitivity Thresholds for CRC and HRA Detection Among Patients with Signs or Symptoms**  **(Sequential Models for CRC and Residual ACNs)** | | | | |
| --- | --- | --- | --- | --- |
| **Performance Characteristic** | **Sensitivity of CRC Detection (Model #1)** | | | **Sensitivity of CRC or HRA Detection in Residual Cohort (Model #2)** |
|  | **100%** | **99%** | **95%** |  |
| % missed CRC | 0 | 0 | 0 | **80%** |
| % missed HRA | 3.5 | 10.7 | 13.8 |  |
| % colonoscopies potentially avoided | 13.1 | 21.8 | 30.5 |  |
| % missed CRC | 0 | 0 | 0 | **70%** |
| % missed HRA | 5.4 | 16.2 | 20.4 |  |
| % colonoscopies potentially avoided | 18.3 | 31.5 | 38.7 |  |
| % missed CRC | 0 | 0 | 0 | **60%** |
| % missed HRA | 7.1 | 21.2 | 27.2 |  |
| % colonoscopies potentially avoided | 21.0 | 37.6 | 46.7 |  |

Example of Interpretation (100% Column):

At 100% sensitivity threshold for CRC detection in Model #1 (entire cohort) and 80% sensitivity for CRC or HRA detection in Model #2 (residual cohort), the sequential modelling strategy would lead to a miss rate of 0% for CRC and 3.5% for HRA while permitting avoidance of up to 13.1% of colonoscopies
