# Supplementary material for: Multivariable models for advanced colorectal neoplasms in screen-eligible individuals at low-to-moderate risk of colorectal cancer: towards improving colonoscopy prioritization
Source: BMC Gastroenterol. 2021 Oct 18;21:383. doi: 10.1186/s12876-021-01965-5 (PMC8524805; doi:10.1186/s12876-021-01965-5)
Supplement: Supplementary file 6 — Additional file 6. Table S6. Model performance at different sensitivity thresholds for CRC detection among patients aged 50–74 (CRC model only). [file 12876_2021_1965_MOESM6_ESM.docx]

| **Supplemental Table 6. Model Performance at Different Sensitivity Thresholds for CRC Detection Among Patients Aged 50 to 74**  **(CRC Model Only)** | | | |
| --- | --- | --- | --- |
| **Performance Characteristic** | **Sensitivity of CRC Detection** | | |
|  | **100%** | **99%** | **95%** |
| % missed CRC | 0 | 0.95 | 4.8 |
| % missed HRA | 37.3 | 41.6 | 62.2 |
| % colonoscopies potentially avoided | 48.8 | 54.1 | 74.5 |

Example of Interpretation (100% Column):

At 100% sensitivity threshold for CRC detection, application of the primary model for CRC would lead to a miss rate of 0% for CRC and 37.3% for HRA while permitting avoidance of up to 48.8% of colonoscopies
