# Supplementary material for: Multivariable models for advanced colorectal neoplasms in screen-eligible individuals at low-to-moderate risk of colorectal cancer: towards improving colonoscopy prioritization
Source: BMC Gastroenterol. 2021 Oct 18;21:383. doi: 10.1186/s12876-021-01965-5 (PMC8524805; doi:10.1186/s12876-021-01965-5)
Supplement: Supplementary file 7 — Additional file 7. Table S7. Model performance at different sensitivity thresholds for CRC and HRA detection among patients aged 50–74 (sequential models for CRC and residual ACNs). [file 12876_2021_1965_MOESM7_ESM.docx]

| **Supplemental Table 7. Model Performance at Different Sensitivity Thresholds for CRC and HRA Detection Among Patients Aged 50 to 74**  **(Sequential Models for CRC and Residual ACNs)** | | | | |
| --- | --- | --- | --- | --- |
| **Performance Characteristic** | **Sensitivity of CRC Detection (Model #1)** | | | **Sensitivity of CRC or HRA Detection in Residual Cohort (Model #2)** |
|  | **100%** | **99%** | **95%** |  |
| % missed CRC | 0 | 0 | 0 | **80%** |
| % missed HRA | 7.6 | 8.1 | 12.4 |  |
| % colonoscopies potentially avoided | 19.0 | 19.6 | 28.5 |  |
| % missed CRC | 0 | 0.95 | 0.95 | **70%** |
| % missed HRA | 11.1 | 12.3 | 18.6 |  |
| % colonoscopies potentially avoided | 25.4 | 27.4 | 38.2 |  |
| % missed CRC | 0 | 0.95 | 1.9 | **60%** |
| % missed HRA | 15.0 | 16.8 | 24.9 |  |
| % colonoscopies potentially avoided | 30.9 | 33.9 | 46.2 |  |

Example of Interpretation (100% Column):

At 100% sensitivity threshold for CRC detection in Model #1 (entire cohort) and 80% sensitivity for CRC or HRA detection in Model #2 (residual cohort), the sequential modelling strategy would lead to a miss rate of 0% for CRC and 7.6% for HRA while permitting avoidance of up to 19.0% of colonoscopies
